# Association between creatinine-to-body weight ratio and arterial stiffness: a cross-sectional and longitudinal study in the Chinese population

**DOI:** 10.3389/fcvm.2026.1640962

**Published:** 2026-02-18

**Authors:** Yuan Shen, Wenbo Zhang, Xue Tian, Yijun Zhang, Qin Xu, Xue Xia, Chenhao Zheng, Xinsheng Han, Xingquan Zhao, Anxin Wang

**Affiliations:** 1 Department of Neurology, Beijing Tiantan Hospital, Capital Medical University, Beijing, China; 2 China National Clinical Research Center for Neurological Diseases, Beijing Tiantan Hospital, Capital Medical University, Beijing, China; 3 Department of Neurology, Kaifeng Central Hospital, Xinxiang Medical University, Kaifeng, China; 4 Department of Epidemiology, Beijing Neurosurgical Institute, Beijing Tiantan Hospital, Capital Medical University, Beijing, China; 5 Department of Clinical Epidemiology and Clinical Trial, Capital Medical University, Beijing, China; 6 Statistics-Mathematics, Rutgers University–New Brunswick, New-Brunswick, NJ, United States; 7 Henan Key Laboratory of Neuromuscular Pathology, Kaifeng Central Hospital, Kaifeng, China

**Keywords:** arterial stiffness, creatinine, cross-sectional analysis, longitudinal analysis, skeletal muscle mass, weight

## Abstract

Few studies have explored the relationship between the creatinine-to-body weight ratio (Cre/BW) and arterial stiffness. Consequently, this research aimed to investigate the association in the Chinese population. This investigation used data from the Asymptomatic Polyvascular Abnormalities Community (APAC) study, where brachial-ankle pulse wave velocity (baPWV) was measured. After a 2-year follow-up period, 1,651 individuals participated in the longitudinal analysis, among whom 464 people had baPWV ≥1,400 cm/s. Multivariable-adjusted logistic regression was employed to calculate the odds ratios (ORs) and 95% confidence intervals (CIs) for the risk of arterial stiffness. The findings from both the cross-sectional and longitudinal analyses were largely in agreement, with the fully adjusted ORs in the highest tertile of the Cre/BW ratio being 1.36 (95% CI, 1.21–1.64) and 2.09 (95% CI, 1.52–2.87), respectively. Restricted cubic splines (RCS) analyses revealed a J-shaped associated in both cross-sectional and longitudinal studies. Subgroup analysis showed that the associations were stronger in participants younger than 60 years (1.40; 95% CI, 1.15–1.71, *P*_int_ = 0.0481). In the cross-sectional analysis, the optimal cutoff value of the Cre/BW ratio to predict arterial stiffness was 1.25, while in the longitudinal analysis, it was 1.29, with corresponding Youden indices of 6.7% and 11%. The study suggested that the Cre/BW ratio was positively associated with the risk of arterial stiffness in Chinese adults.

## Introduction

In recent years, according to research statistics, the prevalence and death rates associated with cardiovascular disease (CVD) have increased significantly, and arterial stiffness represents a significant causative factor and key predictor of CVD (1, 2). Accordingly, early recognition of arterial stiffness is crucial for preventing CVD. Various methods have been proposed to assess arterial stiffness, including the widely used method of measuring brachial-ankle pulse wave velocity (baPWV), which is time-efficient, technically simple, and reliable (3–5). To date, baPWV has been proven to be a valuable marker for the assessment of cardiovascular risk, particularly in Asian populations (6).

In individuals with normal renal function and stable skeletal muscle mass, the rate of creatinine (Cre) excretion is relatively stable (7). Serum Cre is considered an inexpensive and readily available surrogate for assessing skeletal muscle mass (8). Skeletal muscle, as a primary insulin target organ in the human body, plays a key role in coordinating the maintenance of normal blood glucose levels through the uptake and utilization of glucose. A reduction in skeletal muscle mass has been shown to elevate insulin resistance (9, 10), which could lead to metabolic abnormalities including type 2 diabetes (11), metabolic syndrome (11, 12), and atherosclerotic cardiovascular disease (13). Previous studies have identified an association between decreased skeletal muscle mass index and elevated arterial stiffness risk (14). Nevertheless, evidence connecting muscle mass indicators to vascular health remains controversial. Some studies have suggested a protective role of muscle mass (15), while longitudinal epidemiological evidence has demonstrated a positive correlation between higher lean body mass and arterial stiffness (16), potentially influenced by factors such as age, metabolic status, and renal function.

Previous research has utilized the weight-adjusted creatinine-to-body weight ratio (Cre/BW) as a surrogate marker for muscle mass, demonstrating associations with diabetes incidence and all-cause mortality (17, 18). Notably, serum creatinine levels are also influenced by glomerular filtration rate and renal vascular status. When renal function is normal, elevated creatinine levels may partially reflect changes in metabolic load. The relationship between Cre/BW ratio and arterial stiffness remains unclear, and most prior studies have been cross-sectional in design, limiting causal inference. Therefore, we employed data from the Asymptomatic Polyvascular Abnormalities Community (APAC) study to investigate the association between Cre/BW ratio and arterial stiffness risk in the Chinese population, using both cross-sectional and longitudinal analyses.

## Materials and methods

### Study population

**Figure 1:**
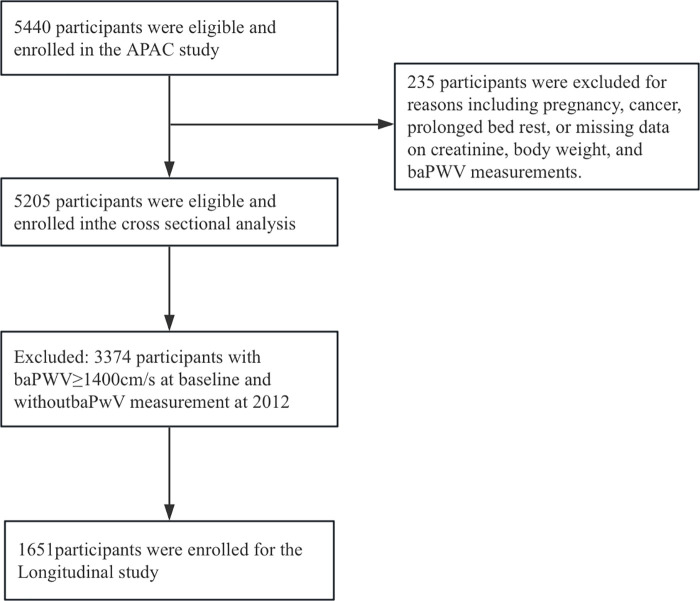
The study's flow diagram.

This cohort study was conducted within the APAC study, based in the Kailuan community of Tangshan City, Hebei Province, China. Eligible participants were aged 40–80 years and underwent standardized questionnaires and physical examinations. We initially recruited 5,440 participants in 2010. We then excluded 235 individuals due to incomplete data on Cre, weight, and baPWV measurements at baseline. To ensure the reliability of the ratio and avoid bias from extreme physiological conditions, outliers with implausible body weight or creatinine values (outside the 1st–99th percentiles) were removed to ensure data validity. People who were pregnant, had cancer, or were bedridden were excluded. This adjustment left us with 5,205 participants for the cross-sectional analysis. For the longitudinal cohort analysis, we further excluded 3,374 participants who had baPWV ≥1,400 cm/s at baseline or who lacked baPWV measurements in 2012. As a result, 1,651 individuals were included in the longitudinal cohort analysis to examine the relationship between the Cre/BW ratio and arterial stiffness (Figure 1).

The study was conducted in compliance with the principles of the Declaration of Helsinki and was approved by the Ethics Committees of Kailuan General Hospital and Beijing Tiantan Hospital. All participants provided written informed consent.

### Measurement of the Cre/BW ratio and variables

The Cre/BW ratio was calculated at baseline. The Cre/BW ratio was defined as Cre (mg/dL) divided by the weight (kg) (19). Participants wore light clothing during weight measurements, and Cre levels were measured using the Jaffe kinetic rate technique. Blood samples were collected early in the morning from the antecubital vein for measurement of fasting blood glucose (FBG), total cholesterol (TC), triglycerides (TG), estimated glomerular filtration rate (eGFR), low-density lipoprotein cholesterol (LDL-C), high-density lipoprotein cholesterol (HDL-C), and high-sensitivity C-reactive protein (hs-CRP) (20). Additional baseline information was obtained using questionnaires. Monthly household income was two classified as <1,000¥, ≥1,000¥, and physical activity was categorized as none, occasional (<80 min/week), or regular (≥80 min/week). Smoking and drinking status was stratified by never, ever, and currently. Body mass index (BMI) was the result of the ratio of weight (kg) to the square of height (m). Blood pressure was measured in the sitting position using a mercury sphygmomanometer, and the average of three measurements was recorded as systolic blood pressure (SBP) and diastolic blood pressure (DBP). Hypertension was defined as SBP ≥140 mmHg or DBP ≥90 mmHg, use of antihypertensive medication, or self-reported history of hypertension. Diabetes was defined as FBG levels ≥7.0 mmol/L, use of glucose-lowering medication, or self-reported history of diabetes. Hyperlipidemia was defined as TC levels ≥5.17 mmol/L, use of lipid-lowering medicine, or self-reported history of hyperlipidemia.

### Measurement of baPWV

Brachial-ankle pulse wave velocity was measured utilizing an automatic arteriosclerosis detection device (BP203RPE III; Omron Healthcare Co, Kyoto, Japan). Participants were required to lie on their backs in thin clothing and remain still during the measurement. Cuffs were fitted on both the arm and ankle. For analysis, the higher baPWV value from either side of the body was recorded. The measurement was repeated twice for each participant, and the maximum values from both sides of baPWV were employed for analysis. Prior to measurement, the participants rested for a minimum of 5 min in a dedicated air-conditioned room (24 ℃–26 ℃).

Arterial stiffness was categorized as baPWV ≥1,400 cm/s (21). In sensitivity analyses, atherosclerosis was considered present when baPWV ≥1,800 cm/s.

### Statistical analysis

Participants were classified into three groups according to the tertiles of the Cre/BW ratio. Continuous variables were reported as median values with interquartile ranges (IQRs), while categorical variables were summarized as counts and percentages. The Wilcoxon and Kruskal–Wallis tests were used to assess differences among groups regarding continuous variables, while the chi-square test was utilized for categorical variables. Logistic regression models were developed to compute the odds ratios (ORs) and 95% confidence intervals (CIs) to explore the association between Cre/BW ratio and various outcomes in the analyses. Model 1 was unadjusted. Model 2 was adjusted for age, sex, education, BMI, income, smoking status, drinking status, hs-CRP, eGFR, FBG, TC, TG, and other risk factors. The selection of variables was based on known risk factors associated with arterial stiffness, which were incorporated in the model as potential confounding factors. Sensitivity analyses were performed using the alternative definition of arterial stiffness (baPWV ≥1,800 cm/s). To examine the association between the Cre/BW ratio and baPWV as continuous variables, we performed multivariable linear regression, adjusting for the confounding factors included in Model 2. To evaluate the impact of the Cre/BW ratio on arterial stiffness progression, ΔbaPWV was calculated as the difference between baPWV values at follow-up and baseline. Multivariable linear regression analysis was used to investigate the association between the Cre/BW ratio and ΔbaPWV, adjusting for baseline baPWV levels and the confounding factors included in Model 2. Furthermore, we conducted a comprehensive analysis of how the Cre/BW ratio influences outcomes as a continuous variable, using restricted cubic spline (RCS), while controlling for all previously cited covariates. We stratified analyses according to specific baseline characteristics—including age, gender, BMI, eGFR, and hypertension (no and yes)—to investigate potential effect modifications in greater depth in cross-sectional and longitudinal analyses.

Receiver operating characteristic (ROC) curves were generated to determine the area under the curve (AUC). We calculated the Youden index, sensitivity, and specificity of the Cre/BW ratio to assess the predictive value of arterial stiffness.

## Results

### Baseline characteristics in the study

A total of 5,205 participants were included in the cross-sectional analysis, with a median age of 52.45 years (IQR, 45.64–61.52), of which 3,125 (60.04%) were men. Table 1 presents the baseline characteristics of participants categorized by tertiles of the Cre/BW ratio. Compared with those in the lowest Cre/BW ratio, those with a higher Cre/BW ratio tended to be older, male, more affluent, and better educated. The baseline characteristics of participants included and excluded were well balanced, as shown in Supplementary Table S1. Supplementary Table S2 further presents the baseline traits of the 1,651 participants in the longitudinal study, organized by their Cre/BW ratio.

**Table 1:** Baseline characteristics of patients according to Cre/BW ratio tertiles in cross-sectional analysis.

Variables	Total (*n* = 5,205)	T1(<1.06) (*n* = 1,736)	T2 (1.07–1.32) (*n* = 1,734)	T3(≥1.33) (*n* = 1,735)	*P*-value
Age, years	52.45 (45.64–61.52)	51.59 (45.56–57.94)	52.95 (45.89–61.77)	53.38 (45.41–68.12)	<0.0001
Men, *n* (%)	3,125 (60.04)	839 (48.33)	1,063 (61.30)	1,223 (70.49)	<0.0001
Female, *n* (%)	2,080 (39.96)	897 (51.67)	671 (38.70)	512 (29.51)	
BMI, kg/m^2^	24.74 (22.66–27.04)	26.53 (24.49–28.57)	24.39 (22.59–26.42)	23.40 (21.48–25.43)	<0.0001
Body weight, kg	69 (61–75)	74 (65–81)	68 (60–75)	65 (58–70.5)	<0.0001
Income, RMB/m, *n* (%)					
<1,000	1,245 (23.92)	342 (19.70)	359 (20.70)	544 (31.35)	<0.0001
≥1,000	3,960 (76.08)	1,394 (80.30)	1,375 (79.30)	1,191 (68.65)	
Active physical activity, *n* (%)					
No physical activity	2,209 (40.33)	712 (41.01)	694 (40.02)	693 (39.94)	0.4051
Occasional physical activity	1,320 (25.36)	459 (26.44)	426 (24.57)	435 (25.07)	
Regular exercise physical activity	1,786 (34.31)	565 (32.55)	614 (35.41)	607 (34.99)	
SBP, mmHg	130.00 (120.00–141.33)	130.00 (120.00–140.67)	130.00 (120.00–140.67)	130.00 (120.00–143.33)	0.1003
DBP, mmHg	80.67 (76.67–90.00)	80.67 (77.33–90.00)	80.67 (76.00–90.00)	80.67 (76.00–90.00)	0.5217
Education, *n* (%)					
Primary school or below	64 3 (12.35)	166 (9.56)	193 (11.13)	284 (16.37)	<0.0001
Middle school	2,306 (44.30)	730 (42.05)	730 (42.10)	846 (48.76)	
High school or above	2,256 (44.30)	840 (48.39)	811 (46.77)	605 (34.87)	
Current smoking, *n* (%)	1,669 (32.07)	462 (26.61)	566 (32.64)	641 (36.95)	<0.0001
Current drinking, *n* (%)	1,716 (32.97)	540 (31.11)	582 (33.56)	594 (34.24)	0.1185
Fasting blood glucose, mmol/L	5.21 (4.82–5.80)	5.30 (4.86–5.92)	5.22 (4.82–5.80)	5.15 (4.80–5.70)	<0.0001
Total cholesterol, mmol/L	4.95 (4.36–5.64)	5.08 (4.46–5.75)	5.00 (4.40–5.66)	4.80 (4.25–5.46)	<0.0001
Triglyceride, mmol/L	1.30 (0.94–1.92)	1.52 (1.04–2.35)	1.29 (0.93–1.87)	1.17 (0.86–1.59)	<0.0001
Hypertension, *n* (%)	1,334 (25.63)	527 (30.36)	419 (24.16)	388 (22.36)	<0.0001
Diabetes, *n* (%)	405 (7.78)	180 (10.37)	114 (6.57)	111 (6.40)	<0.0001
Dyslipidemia, *n* (%)	581 (11.16)	282 (16.24)	177 (10.21)	122 (7.03)	<0.0001
LDL-C, mmol/L	2.60 (2.16–3.05)	2.60 (2.12–3.04)	2.63 (2.19–3.11)	2.63 (2.19–3.00)	0.0223
HDL-C, mmol/L	1.57 (1.30–1.90)	1.59 (1.30–1.92)	1.59 (1.32–1.92)	1.52 (1.30–1.85)	0.0232
Hs-CRP, mg/L	1.00 (0.50–2.15)	1.20 (0.70–2.50)	1.00 (0.50–2.12)	0.89 (0.39–2.00)	<0.0001
eGFR, mL/min/1.73 m^2^	94.50 (80.60–104.51)	104.79 (98.25–110.46)	95.44 (87.00–102.94)	75.87 (66.11–86.74)	<0.0001
Antihypertensive agents, *n* (%)	1,009 (19.39)	398 (22.93)	311 (17.94)	300 (17.29)	<0.0001
Antidiabetic agents, *n* (%)	316 (6.07)	142 (8.18)	88 (5.07)	86 (4.96)	<0.0001
Lipid-lowering agents, *n* (%)	70 (1.34)	39 (2.25)	16 (0.92)	15 (0.86)	0.0003
Creatinine, mg/dL	0.81 (0.70–0.95)	0.68 (0.60–0.76)	0.80 (0.72–0.88)	1.01 (0.88–1.51)	<0.0001
BaPWV, cm/s	1,494 (1,307–1,773)	1,456 (1,282–1,706.50)	1,497 (1,306–1,759)	1,521 (1,327–1,891)	<0.0001
Cre/BW	1.19 (1.01–1.41)	0.94 (0.85–1.01)	1.19 (1.13–1.35)	1.52 (1.41–1.71)	<0.0001

BMI, body mass index; DBP, diastolic blood pressure; SBP, systolic blood pressure; LDL-C, low-density lipoprotein cholesterol; HDL-C, high-density lipoprotein cholesterol; hs-CRP, high-sensitivity C-reactive protein; eGFR, estimated glomerular filtration rate; BaPWV, brachial-ankle pulse wave velocity; Cre/BW, Cre-to-body weight ratio, calculated as serum creatinine (mg/dL) divided by body weight (kg) multiplied by 100.

### Cross-sectional analysis

**Figure 2:**
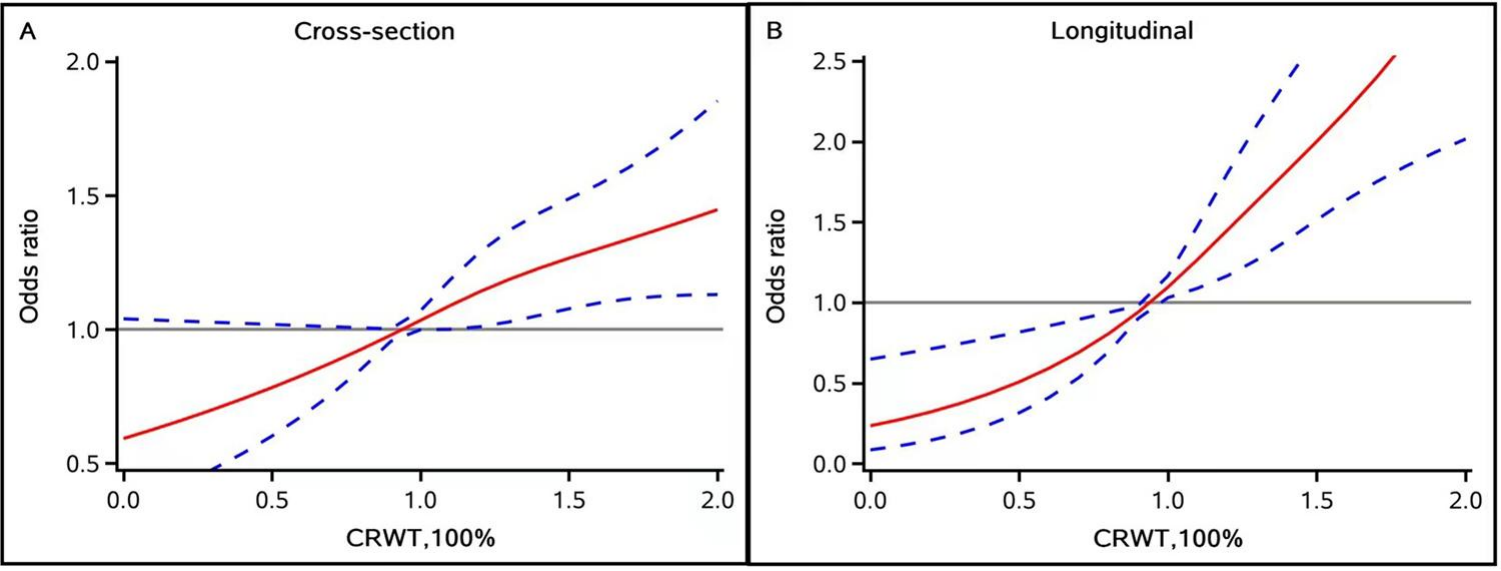
Multivariable-adjusted association of the Cre/BW ratio and baPWV≥1,400 cm/s based on restricted cubic spines with four knots at 5th, 35th, 65th, and 95th percentiles of Cre/BW ratio. Adjusted for age, sex, education, body mass index, income, smoking status, drinking status, high-sensitivity C-reactive protein, estimated glomerular filtration rate, fasting blood glucose, total cholesterol, triglyceride, history of hypertension diabetes, dyslipidemia, antihypertensive, antidiabetic, and lipid-lowering agents. **(A)** Cross-sectional analysis RCS. **(B)** Longitudinal analysis RCS.

A total of 3,227 participants exhibited baPWV measurements exceeding 1,400 cm/s. Table 2 shows the Cre/BW ratio tertiles with the prevalence of arterial stiffness. The prevalence increased with higher Cre/BW tertiles. In Model 1, the highest tertile was significantly associated with arterial stiffness (OR: 1.35; 95% CI, 1.18–1.55; *P* < 0.0001). This association remained significant after multivariable adjustment (Model 2 OR: 1.36; 95% CI, 1.21–1.64; *P* < 0.0001). Similar trends were observed in the sensitivity analysis (OR: 1.39; 95% CI, 1.10–1.77; *P* = 0.0056). The multivariate-adjusted curvilinear regression results showed a J-shaped association when baPWV was evaluated as a continuous variable (Figure 2).

**Table 2:** ORs (95% CIs) for the cross-sectional and longitudinal association of the Cre/BW ratio tertiles with the prevalence of baPWV≥1,400 cm/s.

Analysis	Outcomes	T1 (<1.06)	T2 (1.07–1.32)	T3 (≥1.33)	Per 1 unit increase	*P* for trend
Cross-sectional association of the Cre/BW ratio	BaPWV≥1,400 cm/s, *n* (%)	1,012 (58.29)	1,081 (62.34)	1,134 (65.36)		<0.0001
	Model 1	Reference	1.18 (1.03–1.36)	1.35 (1.18–1.55)	1.45 (1.22–1.72)	<0.0001
	Model 2	Reference	1.23 (1.03–1.46)	1.36 (1.21–1.64)	1.49 (1.17–1.91)	<0.0001
	Sensitivity analysis	Reference	1.16 (0.94–1.46)	1.39 (1.10–1.77)	1.49 (1.17–1.91)	0.0056
Longitudinal association of the Cre/BW ratio	BaPWV≥1,400 cm/s, *n* (%)	138 (22.70)	164 (29.56)	162 (33.13)		0.0005
	Model 1	Reference	1.43 (1.10–1.86)	1.68 (1.29–2.20)	2.20 (1.56–3.09)	0.0001
	Model 2	Reference	1.59 (1.19–2.12)	2.09 (1.52–2.87)	3.03 (2.02–4.54)	<0.0001
	Sensitivity analysis	Reference	1.10 (0.81–1.47)	1.27 (0.92–1.74)	3.03 (2.02–4.54)	0.1436

baPWV, branchial-ankle pulse wave velocity.

Model 1: unadjusted.

Model 2: adjusted for age, sex, education, body mass index, income, smoking status, drinking status, high-sensitivity C-reactive protein, estimated glomerular filtration rate, fasting blood glucose, total cholesterol, triglyceride, history of hypertension diabetes, dyslipidemia, antihypertensive, antidiabetic, and lipid-lowering agents.

Sensitivity analysis was performed by redefining baPWV≥1,800 cm/s and adjusting variables in Model 2.

### Longitudinal analysis

Table 2 presents the ORs (95% CIs) in the longitudinal relationship with different tertiles of the Cre/BW ratio. We observed an increasing risk of incident arterial stiffness across increasing Cre/BW tertiles. A statistically significant association was observed between the highest Cre/BW ratio tertile and the prevalence of arterial stiffness in unadjusted model (OR: 1.68; 95% CI, 1.29–2.20; *P* = 0.0001). In Model 2, the highest Cre/BW ratio tertiles demonstrated a significantly greater risk of developing arterial stiffness in contrast to the lowest Cre/BW ratio tertiles (OR: 2.09; 95% CI, 1.52–2.87; *P* < 0.0001). Sensitivity analysis also confirmed similar results. In an additional longitudinal analysis using the change in baPWV (ΔbaPWV) as a continuous outcome, a higher Cre/BW ratio was associated with a greater increase in baPWV during follow-up (Supplementary Table S4).

### Association between Cre/BW ratio and continuous baPWV

In multivariable linear regression models treating baPWV as a continuous outcome (Table 3), we observed a significant positive association in both analyses. In the cross-sectional analysis, each 1-unit increase in the Cre/BW ratio was associated with a 36.16 cm/s higher baPWV (*P* < 0.0001). In the longitudinal analysis, each 1-unit increase in Cre/BW ratio was associated with an 84.55 cm/s higher baPWV at follow-up (*P* = 0.0003). These results were consistent with the findings from the logistic regression models.

**Table 3:** Association between Cre/BW ratio and baPWV as a continuous variable using multivariable linear regression analysis.

Analysis	Cre/BW ratio	*β*	95% CI	*P* for trend
Cross-sectional	Per 1 unit increase	36.16	(18.08, 54.24)	<0.0001
Longitudinal	Per 1 unit increase	84.55	(39.36, 129.74)	0.0003

Adjusted for age, sex, education, body mass index, income, smoking status, drinking status, high-sensitivity C-reactive protein, estimated glomerular filtration rate, fasting blood glucose, total cholesterol, triglyceride, history of hypertension diabetes, dyslipidemia, antihypertensive, antidiabetic, and lipid-lowering agents.

### Subgroup analyses

To minimize the potential confounding effect of renal function on the Cre/BW ratio, we performed a stratified analysis based on eGFR levels (Table 4). In the longitudinal analysis, participants with normal renal function (eGFR ≥90 mL/min/1.73 m^2^) in the highest tertile of the Cre/BW ratio had a significantly increased risk of developing arterial stiffness compared with those in the lowest tertile (OR: 2.06; 95% CI: 1.21–3.49; *P* = 0.004). Table 5 and Supplementary Table S3 present the subgroup analysis data, demonstrating that the association between Cre/BW ratio and the risk of arterial stiffness was consistent across various subgroups. In the cross-sectional analysis (Table 5), we observed a more robust association between Cre/BW ratio and the risk of incident arterial stiffness among participants younger than 60 years (1.40; 95% CI, 1.15–1.71; *P* = 0.0481).

**Table 4:** Association between Cre/BW ratio tertiles and risk of baPWV≥1,400 cm/s stratified by eGFR categories.

Analysis	eGFR (mL/min/1.73 m^2^)	T1 (<1.06)	T2 (1.07–1.32)	T3 (≥1.33)	*P* for trend
Cross-sectional association of the Cre/BW ratio	eGFR < 90	Reference	1.72 (0.91–3.28)	1.98 (1.01–3.87)	0.13
	eGFR ≥ 90	Reference	1.25 (1.02–1.53)	1.27 (0.91–1.77)	0.09
Longitudinal association of the Cre/BW ratio	eGFR < 90	Reference	1.19 (0.35–3.99)	1.47 (0.41–5.34)	0.70
	eGFR ≥ 90	Reference	1.70 (1.21–2.40)	2.06 (1.21–3.49)	0.004

Adjusted for age, sex, education, body mass index, income, smoking status, drinking status, high-sensitivity C-reactive protein, estimated glomerular filtration rate, fasting blood glucose, total cholesterol, triglyceride, history of hypertension diabetes, dyslipidemia, antihypertensive, antidiabetic, and lipid-lowering agents.

**Table 5:** Association between Cre/BW ratio and baPWV≥1,400 cm/s in the cross-sectional stratified subgroups.

Subgroup	OR (95% CIs)			*P* for interaction
	T1 (<1.06)	T2 (1.07–1.32)	T3 (≥1.33)	
Sex				0.7993
Men	Reference	1.16 (0.92–1.46)	1.24 (0.97–1.59)	
Female	Reference	1.41 (1.07–1.86)	1.60 (1.13–2.13)	
Age	Reference			0.0481
<60 years	Reference	1.27 (1.06–1.52)	1.40 (1.15–1.71)	
≥60 years	Reference	0.75 (0.38–1.47)	0.59 (0.29–1.20)	
BMI				0.7434
<24 kg/m^2^	Reference	1.30 (0.94–1.79)	1.33 (0.96–1.85)	
≥24 kg/m^2^	Reference	1.20 (0.97–1.48)	1.38 (1.09–1.76)	
eGFR				0.9670
<90 mL/min/1.73 m^2^	Reference	1.23 (1.06–1.47)	1.23 (1.03–1.46)	
≥90 mL/min/1.73 m^2^	Reference			
Hypertension				0.4697
Yes	Reference	0.86 (0.55–1.33)	1.19 (0.67–2.11)	
No	Reference	1.30 (1.04–1.57)	1.40 (1.14–1.71)	
Diabetes				0.5646
Yes	Reference	1.06 (0.46–2.44)	1.07 (0.41–2.78)	
No	Reference	1.26 (1.05–1.50)	1.38 (1.13–1.68)	
Dyslipidemia				0.5041
Yes	Reference	1.28 (0.72–2.25)	1.04 (0.51–2.14)	
No	Reference	1.21 (1.01–1.46)	1.40 (1.14–1.70)	

BMI, body mass index; eGFR, estimated glomerular filtration rate.

Adjusted for age, sex, education, body mass index, income, smoking status, drinking status, high-sensitivity C-reactive protein, estimated glomerular filtration rate, fasting blood glucose, total cholesterol, triglyceride, history of hypertension diabetes, dyslipidemia, antihypertensive, antidiabetic, and lipid-lowering agents.

### Predictive ability of the Cre/BW ratio

**Figure 3:**
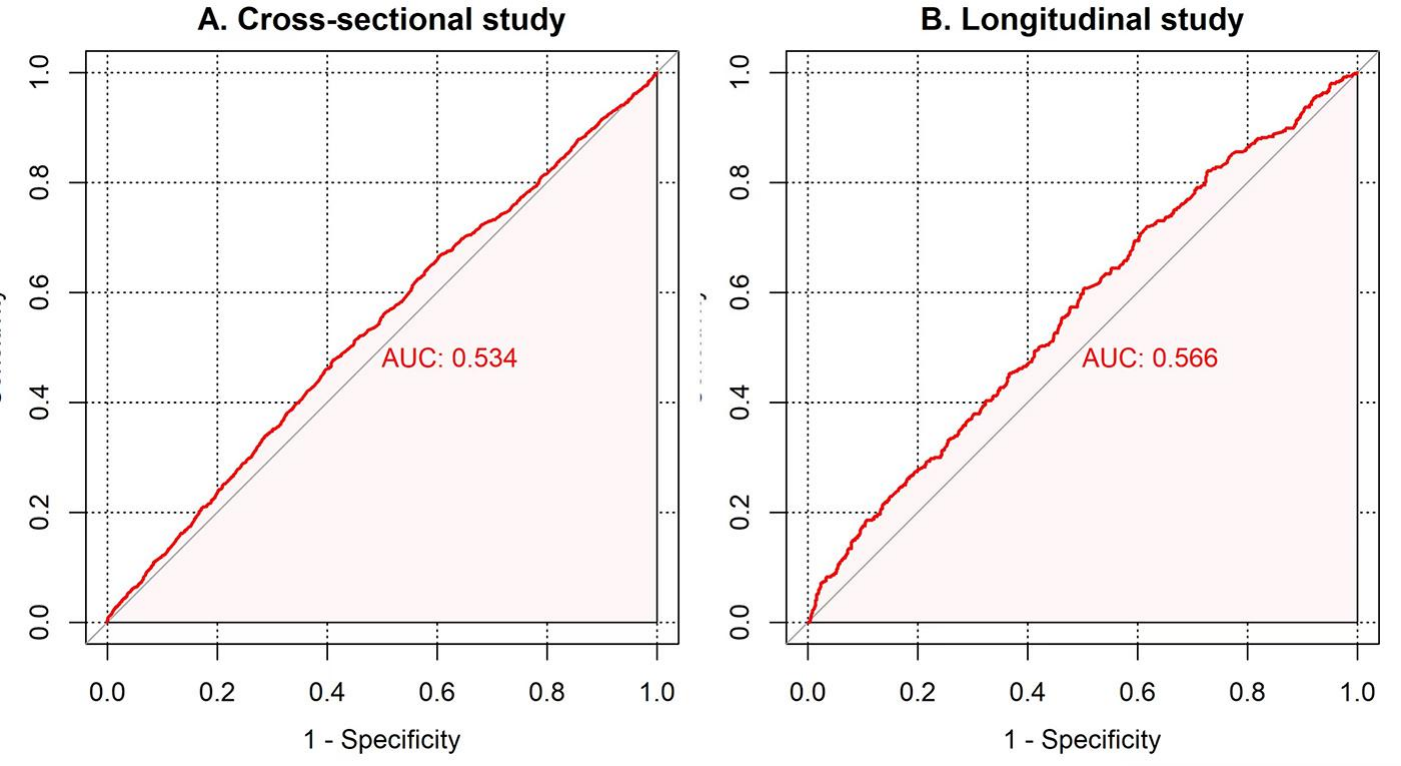
The ROC curves according to the Cre/BW ratio with baPWV ≥1,400 cm/s. AUC, area under the curve; 95% CI, 95% confidence interval. **(A)** ROC curve of cross-sectional analysis. **(B)** ROC curve of longitudinal analysis.

In the cross-sectional study, ROC curve evaluation identified 1.25 as the best cutoff value to predict the occurrence of arterial stiffness. This yielded an AUC of 0.534 (95% CI: 0.52–0.55, *p* < 0.0001), a sensitivity of 47.8%, a specificity of 58.9%, and a Youden index of 6.7% (Figure 3). Furthermore, the AUC for the Cre/BW ratio in longitudinal research is 0.566 (95% CI: 0.54–0.60, *p* < 0.0001) and the optimal cutoff value is 1.29 (sensitivity is 61%, specificity is 50%, and Youden index is 11%).

## Discussion

In this prospective cohort study conducted in China, we confirmed a significant positive correlation between the Cre/BW ratio and arterial stiffness risk, evident in both cross-sectional and longitudinal assessments. Our analysis revealed a J-shaped relationship between the Cre/BW ratio and the risk of arterial stiffness. Subgroup analysis revealed that this correlation was more prominent in younger adults. In those aged 60 years and older, the Cre/BW ratio appeared to be a protective factor. This discrepancy likely reflects the shifting physiological significance of the Cre/BW ratio with aging. In older adults, a decline in Cre/BW ratio is indicative of sarcopenia, which accelerates vascular aging. In contrast, among middle-aged adults, where sarcopenia is less common, a higher Cre/BW ratio may indicate increased metabolic burden or renal stress.

Our findings align closely with emerging evidence linking higher muscle mass to increased arterial stiffness. A longitudinal study focusing on adolescents indicated that lean mass, assessed through dual-energy X-ray absorptiometry, was strongly associated with the progression of arterial stiffness over 7 years (16). Similarly, a cross-sectional study demonstrated that fat-free mass index was positively associated with arterial stiffness in middle-aged adults, identifying it as an independent predictor of vascular risk after adjustment for confounders (22). However, in contrast to the above findings, studies using bioelectrical impedance analysis to measure skeletal muscle mass reported that skeletal muscle loss is associated with the progression of arterial stiffness (23, 24). Furthermore, an analysis of 5,566 participants found no association between lean muscle mass (measured by dual-energy X-ray absorptiometry) and arterial stiffness (25). This discrepancy in findings may suggest that the physiological impact of muscle mass on vascular health is highly complex. While skeletal muscle has traditionally been regarded as protective, in certain contexts, indicators such as the Cre/BW ratio may reflect the adverse effects of metabolic burden rather than solely the benefits of muscle mass.

Discrepancies in previous research findings may stem from methodological differences. First, increases in skeletal muscle mass are often accompanied by rising body fat (26, 27), a potential confounding factor that early studies using simple creatinine markers may have overlooked. Second, with advancing age, older adults appear to experience both muscle mass reduction and body fat redistribution, leading to biased outcomes. Furthermore, lean body mass encompasses not only skeletal muscle mass but also metabolically active visceral organs (28). Therefore, the positive correlation observed may reflect overall metabolic load rather than solely the influence of skeletal muscle. In subgroup analyses, we found age to be a key interacting factor, with elevated Cre/BW ratios associated with increased arterial stiffness risk among adults under 60 years of age. Previous studies also suggested that in individuals under 55 years of age, higher aortic pulse wave velocity is positively correlated with lower creatinine clearance (29), potentially due to reduced creatinine levels resulting from skeletal muscle loss and body composition changes during aging. Unlike prior subgroup studies, our analysis did not observe an interaction effect for gender (30).

Given that the glomerular filtration rate affects serum creatinine levels, we further stratified the analysis by eGFR. Longitudinal analysis results suggested that in participants with normal renal function, the Cre/BW ratio remained positively correlated with arterial stiffness, whereas this association was weaker among those with reduced eGFR, possibly due to the smaller sample size. Longitudinal analysis reflected the risk of arterial stiffening over time, thereby enhancing the robustness of the association. At present, the biological mechanisms linking a higher Cre/BW ratio to increased arterial stiffness have not been fully elucidated. The following hypothetical mechanisms are proposed as explanations: First, skeletal muscle and other lean body tissues, as metabolically active components, often increase alongside higher demands for circulating blood volume and cardiac output. This imposes a hemodynamic load on arterial walls, inducing arterial stiffness (31, 32). Second, when elevated metabolic demands are reflected in renal function, they may manifest as increased creatinine production. Even within the normal renal function range, a mild elevation in creatinine may indicate early microvascular or endothelial dysfunction. Microvascular injury can coexist with endothelial dysfunction and arterial stiffness (33). Finally, elevated metabolic load induces oxidative stress and chronic inflammation, stimulating the renin–angiotensin system and further accelerating vascular wall remodeling (34).

The strength of this study lies in its utilization of a combined cross-sectional and longitudinal design, which allowed us to explore the correlation between Cre/BW ratio and the risk of arterial stiffness, which yielded significant results. Sensitivity analyses further corroborated our findings. Nevertheless, several limitations in our research should be acknowledged. The relatively low AUC values in our ROC analysis indicate that the Cre/BW ratio has limited utility as a diagnostic tool for arterial stiffness. Future studies may develop integrated models incorporating multiple risk factors alongside the Cre/BW ratio, which are expected to significantly enhance predictive performance. In addition, our study population was recruited from the Kailuan community, which may lead to limitations in the global validity of the findings due to potential influence of racial and regional differences. Further analysis of the Cre/BW ratio for various populations is required. Moreover, this study has a large sample size, which inevitably resulted in a high rate of loss to follow-up in the longitudinal study. Furthermore, the possibility of drug interference with Cre levels cannot be entirely discounted. Finally, we lack information on body composition in the baseline profile, including details on muscle mass and body fat mass, which precluded further analysis. The availability of such data would allow more precise comparison of the Cre/BW ratio with the skeletal muscle mass.

## Conclusions

In conclusion, our study demonstrated that an elevated Cre/BW ratio was associated with an increased risk of developing arterial stiffness. This trend was more pronounced among adults younger than 60 years. The results indicated that screening Cre/BW could effectively pinpoint individuals who are particularly susceptible to arterial stiffness.

## Data Availability

The datasets used and analyzed during the current study are available from the corresponding author upon reasonable request. Requests to access these datasets should be directed to wanganxin@bjtth.org.
