# Supplementary material for: Association between creatinine-to-body weight ratio and arterial stiffness: a cross-sectional and longitudinal study in the Chinese population
Source: Front Cardiovasc Med. 2026 Feb 18;13:1640962. doi: 10.3389/fcvm.2026.1640962 (PMC12957206; doi:10.3389/fcvm.2026.1640962)
Supplement: Supplementary file 1 [file Datasheet1.docx]

**Table S1** Baseline characteristics of patients included versus excluded in this study

| Variables | Total  (n=5440) | Exclude  (n=235) | Include  (n=5205) | *P* value |
| --- | --- | --- | --- | --- |
| Age, years | 52.48(45.65-61.58) | 52.58(45.90-63.26) | 52.45(45.64-61.52) | 0.4256 |
| Men, n (%) | 3257(59.87) | 132(56.17) | 3125(60.04) | 0.2367 |
| Female, n (%) | 2183(40.13) | 103(43.83) | 2080(39.96) |  |
| BMI, kg/m^2^ | 24.74(22.66-27.04) | 24.56(22.66-27.31) | 24.74(22.66-27.04) | 0.9613 |
| Income, RMB/m, n (%) |  |  |  | 0.7890 |
| <1000 | 1303(23.95) | 58(24.68) | 1245(23.92) |  |
| ≥1000 | 4137(76.05) | 177(75.32) | 3960(76.08) |  |
| Active physical activity, n (%) |  |  |  | 0.1167 |
| No physical activity | 2178(40.04) | 79(33.62) | 2099(40.33) |  |
| Occasional physical activity | 1388(25.51) | 68(28.94) | 1320(25.36) |  |
| Regular exercise physical activity | 1874(34.45) | 88(37.45) | 1786(34.31) |  |
| SBP, mmHg | 130.00(120.00-140.67) | 129.33(72.00-88.67) | 130.00(120.00-141.33) | 0.0221 |
| DBP, mmHg | 80.67 (76.67-90.00) | 80.00(72.00-88.00) | 80.67(76.67-90.00) | 0.0358 |
| Education, n (%) |  |  |  | 0.0041 |
| Primary school or below | 663(12.19) | 20(8.51) | 643(12.35) |  |
| Middle school | 2394(44.01) | 88(37.45) | 2306(44.30) |  |
| High school or above | 2383(43.81) | 127(54.04) | 2256(43.34) |  |
| Current smoking n (%) | 1737(31.93) | 68(28.94) | 1669(32.07) | 0.3142 |
| Current drinking n (%) | 1799(32.07) | 83(35.32) | 1716(32.97) | 0.4537 |
| Fasting blood glucose, mmol/L | 5.21(4.82-5.80) | 5.19(4.86-5.52) | 5.21(4.82-5.5.80) | 0.2469 |
| Total cholesterol, mmol/L | 4.95(4.36-5.63) | 4.90(4.35-5.60) | 4.95(4.36-5.64) | 0.6888 |
| Triglyceride, mmol/L | 1.30(0.93-1.93) | 1.31(0.89-2.00) | 1.30(0.93-1.92) | 0.8487 |
| Hypertension, n (%) | 1393(25.61) | 59(25.11) | 1334(25.63) | 0.8575 |
| Diabetes, n (%) | 424(7.79) | 19(8.09) | 405(7.78) | 0.8649 |
| Dyslipidemia, n (%) | 616(11.32) | 35(14.89) | 581(11.16) | 0.0775 |
| LDL-C, mmol/L | 2.60(2.16-3.05) | 2.60(2.12-3.04) | 2.63(2.19-3.11) | 0.7017 |
| HDL-C, mmol/L | 1.57(1.30-1.90) | 1.63(1.34-1.87) | 1.57(1.30-1.90) | 0.4002 |
| Hs-CRP, mg/L | 1.00(0.50-2.20) | 1.00(0.56-2.27) | 1.00(0.50-2.20) | 0.4473 |
| eGFR, ml/min/1.73m^2^ | 94.54(80.77-104.59) | 96.75(84.78-106.53) | 94.50(80.60-104.51) | 0.0239 |
| Antihypertensive agents, n (%) | 1052(19.34) | 43(18.30) | 1009(19.39) | 0.6797 |
| Antidiabetic agents, n (%) | 328(6.03) | 12(5.11) | 316(6.07) | 0.5434 |
| Lipid-lowering agents, n (%) | 75(1.38) | 5(2.13) | 70(1.34) | 0.3141 |

Abbreviations: BMI, body mass index, DBP diastolic blood pressure, SBP systolic blood pressure, LDL-C low-density lipoprotein cholesterol, HDL-C high-density lipoprotein cholesterol, hs-CRP high-sensitivity C-reactive protein, eGFR estimated glomerular filtration rate.

**Table S2** baseline characteristics of patients according to Cre/BW ratio tertiles in longitudinal

| Variables | Total  (n=1651) | T1(<1.06)  (n=607) | T2(1.07-1.32)  (n=555) | T3(≥1.33)  (n=489) | *P* value |
| --- | --- | --- | --- | --- | --- |
| Age, years | 46.12(42.96-51.54) | 46.32(43.20-51.55) | 46.52(43.06-51.97) | 45.26(42.70-50.63) | <0.0001 |
| Men, n (%) | 745(45.12) | 221(36.41) | 257(46.31) | 267(54.60) | <0.0001 |
| BMI, kg/m^2^ | 24.38(22.43-26.53) | 25.71(23.74-27.68) | 24.15(22.05-26.06) | 23.11(21.48-24.91) | <0.0001 |
| Body weight, kg | 67(60-75) | 70(64-80) | 65(60-75) | 63(56-70) | <0.0001 |
| Income ≥ 1000, RMB/m, n (%) | 1190(72.50) | 472(77.76) | 417(75.14) | 308(62.99) | <0.0001 |
| Active physical activity, n (%) | 480(29.07) | 183(30.15) | 164(29.55) | 133(27.20) | 0.8084 |
| No physical activity | 725(43.91) | 258(42.50) | 243(43.78) | 224(45.81) |  |
| Occasional physical activity | 480(29.07) | 183(30.15) | 164(29.55) | 133(27.20) |  |
| Regular exercise physical activity | 446(27.01) | 166(27.35) | 148(26.67) | 132(26.99) |  |
| SBP, mmHg | 120.00(110.00-130.00) | 119.33(109.33-130.00) | 120.00(110.00-130.00) | 120.00(110.00-139.33) | 0.3967 |
| DBP, mmHg | 80.00(70.67-84.00) | 80.00(70.00-83.33) | 80.00(70.67-84.00) | 80.00(72.00-86.00) | 0.0187 |
| Education, n (%) |  |  |  |  | <0.0001 |
| Primary school or below | 69(4.18) | 23(3.79) | 22(3.96) | 24(4.91) |  |
| Middle school | 710(43.00) | 227(37.40) | 228(41.08) | 255(52.15) |  |
| High school or above | 872(52.82) | 357(58.81) | 305(54.95) | 210(42.94) |  |
| Current smoking n (%) | 453(27.44) | 129(21.25) | 157(28.29) | 167(34.15) | <0.0001 |
| Current drinking n (%) | 446(27.01) | 155(25.54) | 144(25.95) | 147(30.06) | 0.1923 |
| Fasting blood glucose, mmol/L | 5.07(4.71-5.50) | 5.06(4.70-5.50) | 5.07(4.73-5.51) | 5.08(4.70-5.50) | 0.6314 |
| Total cholesterol, mmol/L | 4.80(4.26-5.45) | 4.88(4.32-5.56) | 4.83(4.27-5.52) | 4.69(4.20-5.12) | <0.0001 |
| Triglyceride, mmol/L | 1.16(0.83-1.71) | 1.31(0.90-2.07) | 1.12(0.81-1.62) | 1.07(0.79-1.41) | <0.0001 |
| Hypertension, n (%) | 126(7.63) | 70(11.53) | 42(7.57) | 14(2.86) | <0.0001 |
| Diabetes, n (%) | 37(2.24) | 18(2.97) | 15(2.70) | 4(0.82) | 0.0385 |
| Dyslipidemia, n (%) | 129(7.81) | 83(13.67) | 32(5.77) | 14(2.86) | <0.0001 |
| LDL-C, mmol/L | 2.50(2.10-2.90) | 2.44(2.01-2.86) | 2.50(2.14-2.92) | 2.55(2.20-2.90) | 0.0081 |
| HDL-C, mmol/L | 1.63(1.37-1.95) | 1.68(1.41-1.96) | 1.64(1.38-1.97) | 1.57(1.33-1.88) | 0.0122 |
| Hs-CRP, mg/L | 0.78(0.40-1.50) | 0.90(0.50-1.80) | 0.90(0.50-1.80) | 0.54(0.20-1.20) | <0.0001 |
| eGFR, ml/min/1.73m^2^ | 100.60(86.67-107.93) | 107.98(103.70-112.60) | 99.76(90.72-106.05) | 79.06(67.98-91.35) | <0.0001 |
| Antihypertensive agents, n (%) | 83(5.03) | 46(7.57) | 29(5.23) | 8(1.64) | <0.0001 |
| Antidiabetic agents, n (%) | 22(1.33) | 10(1.65) | 10(1.80) | 2(0.41) | 0.1023 |
| Lipid-lowering agents, n (%) | 13(0.79) | 7(1.51) | 4(0.72) | 2(0.41) | 0.3739 |
| Creatinine, mg/dL | 0.78(0.66-0.92) | 0.65(0.58-0.75) | 0.78(0.70-0.87) | 0.98(0/85-1.15) | <0.0001 |
| BaPWV, cm/s | 1258(1179-1329) | 1251(1171-1326) | 1254(1178-1327) | 1271(1193-1333) | 0.055 |
| Cre/BW | 1.16(1.00-1.38) | 0.95(0.86-1.02) | 1.19(1.13-1.24) | 1.53(1.41-1.70) | <0.0001 |

Abbreviations: BMI body mass index, DBP diastolic blood pressure, SBP systolic blood pressure, LDL-C low-density lipoprotein cholesterol, HDL-C high-density lipoprotein cholesterol, hs-CRP high-sensitivity C-reactive protein, eGFR estimated glomerular filtration rate, BaPWV brachial-ankle pulse wave velocity, Cre/BW Cre to body weight ratio, calculated as serum creatinine (mg/dL) divided by body weight (kg) multiplied by 100.

**Table S3** Association between Cre/BW ratio and baPWV≥1400 cm/s in the longitudinal stratified subgroups

| Subgroup |  | OR(95% CIs) |  | *P* for |
| --- | --- | --- | --- | --- |
|  | T1(<1.06) | T2(1.07-1.32) | T3(≥1.33) | interaction |
| Sex |  |  |  | 0.7964 |
| Man | Reference | 1.41(0.90-2.12) | 1.92(1.27-2.97) |  |
| Female | Reference | 1.71(1.12-2.61) | 2.19(1.35-3.56) |  |
| Age | Reference |  |  | 0.4632 |
| < 60years | Reference | 1.53(1.40-2.07) | 2.06(1.49-2.85) |  |
| ≥ 60years | Reference | 2.70(0.44-16.75) | 2.21(0.25-19.92) |  |
| BMI |  |  |  | 0.6653 |
| < 24kg/m^2^ | Reference | 1.35(0.79-2.30) | 2.13(1.25-3.65) |  |
| ≥ 24kg/m^2^ | Reference | 1.67(1.18-2.38) | 1.99(1.32-3.01) |  |
| eGFR | Reference |  |  | * |
| < 90ml/min/1.73m^2^ |  |  |  |  |
| ≥ 90ml/min/1.73m^2^ | Reference | 1.60(1.19-2.13) | 2.01(1.44-2.80) |  |
| Hypertension |  |  |  | 0.9491 |
| Yes | Reference | 1.02(0.38-2.72) | 1.34(0.31-5.88) |  |
| No | Reference | 1.64(1.20-2.23) | 2.17(1.56-3.03) |  |
| Diabetes |  |  |  | 0.1781 |
| Yes | Reference | 6.79(0.08-597.99) |  |  |
| No | Reference | 1.54(1.14-2.07) | 2.03(1.47-2.81) |  |
| Dyslipidemia |  |  |  | 0.0578 |
| Yes | Reference | 1.19(0.43-3.32) | 0.18(0.02-1.59) |  |
| No | Reference | 1.70(1.25-2.31) | 2.35(1.68-3.28) |  |

Abbreviations: BMI body mass index, eGFR estimated glomerular filtration rate.

adjusted for age, sex, education, body mass index, income, smoking status, drinking status, high-sensitivity C-reactive protein, estimated glomerular filtration rate, fasting blood glucose, total cholesterol, triglyceride, history of hypertension diabetes, and dyslipidemia, antihypertensive, antidiabetic, lipid-lowering agents.

**Table S4** Association between the Cre/BW ratio and change in baPWV (Δ baPWV).

|  | β (95% CI) | P value |
| --- | --- | --- |
| Cre/BW ratio (continuous) |  |  |
| Per 1-unit increase | 71.50 (28.04, 114.96) | 0.0013 |
| Cre/BW ratio (tertiles) |  |  |
| T1(<1.06) | Reference |  |
| T2(1.07-1.32) | 30.82(1.36, 60.28) | 0.0403 |
| T3(≥1.33) | 47.80(15.01, 80.59) | 0.0043 |
| P overall |  | 0.014 |

Model was adjusted for age, sex, education, body mass index, income, smoking status, drinking status, high-sensitivity C-reactive protein, estimated glomerular filtration rate, fasting blood glucose, total cholesterol, triglyceride, history of hypertension diabetes, and dyslipidemia, antihypertensive, antidiabetic, lipid-lowering agents.
